# Small extracellular vesicles containing LDLR^Q722*^ protein reconstructed the lipid metabolism via heparan sulphate proteoglycans and clathrin‐mediated endocytosis

**DOI:** 10.1002/ctm2.773

**Published:** 2022-03-28

**Authors:** Yingchao Zhou, Qiang Xie, Silin Pan, Jianfei Wu, Xiangyi Wang, Zhubing Cao, Mengru Wang, Lingfeng Zha, Mengchen Zhou, Qianqian Li, Qing Wang, Xiang Cheng, Gang Wu, Xin Tu

**Affiliations:** ^1^ Key Laboratory of Molecular Biophysics of the Ministry of Education College of Life Science and Technology Center for Human Genome Research Cardio‐X Institute Huazhong University of Science and Technology Wuhan China; ^2^ Heart Center Women and Children's Hospital Qingdao University Qingdao China; ^3^ Department of Cardiology The First Affiliated Hospital of Xiamen University Xiamen China; ^4^ Department of Cardiology Union Hospital Tongji Medical College Huazhong University of Science and Technology Wuhan China; ^5^ Department of Cardiology Renmin Hospital of Wuhan University Wuhan China

Dear Editor,

Familial hypercholesterolemia (FH) is a severe inherited lipid metabolism dysfunction, characterised by high‐total and low‐density lipoprotein (LDL) cholesterol levels.^1^ Mutation of *LDLR*, *PCSK9* and *APOB* are the most common genetic etiology of FH.^2^ Currently, the statins therapy is the mainstay treatment for FH.^3^ However, higher rate of side effect and statin‐induced PCSK9 increase limits statins efficacy of LDL lowering.^4^ As an alternative to the statins therapy, PCSK9 inhibitors treatment is effective in the vast majority of FH patients, but LDLR homozygous deletion patients fail to respond to it.^3^ Therefore, exploring and developing new lipid‐lowering drugs are of great significance.

Up to February 2022, there are 3843 *LDLR* variants in the LOVD FH database, most of which exhibit heterozygosity. Few homozygote mutations have been reported, especially deletion homozygous mutations (Table S1), which account for just 0.67% of all reported mutations. Using whole‐exome sequencing, we identified a homozygote nonsense mutation *LDLR* c.C2164T (p.Q722*) in a consanguineous Chinese FH family (Figure 1A,B, Table S2). The mutation had extremely low frequencies, and was absent from the ExAC database. LDLR is a transmembrane glycoprotein composed of 860 amino acids, which is used for receptor‐mediated endocytosis of LDL.^5^The mutant LDLR translated into a truncated protein (1‐722 amino acids, Figure S1A,B). We referred to this truncated LDLR as “LDLR^Q722*^” to distinguish from the wild‐type LDLR.

**FIGURE 1:**
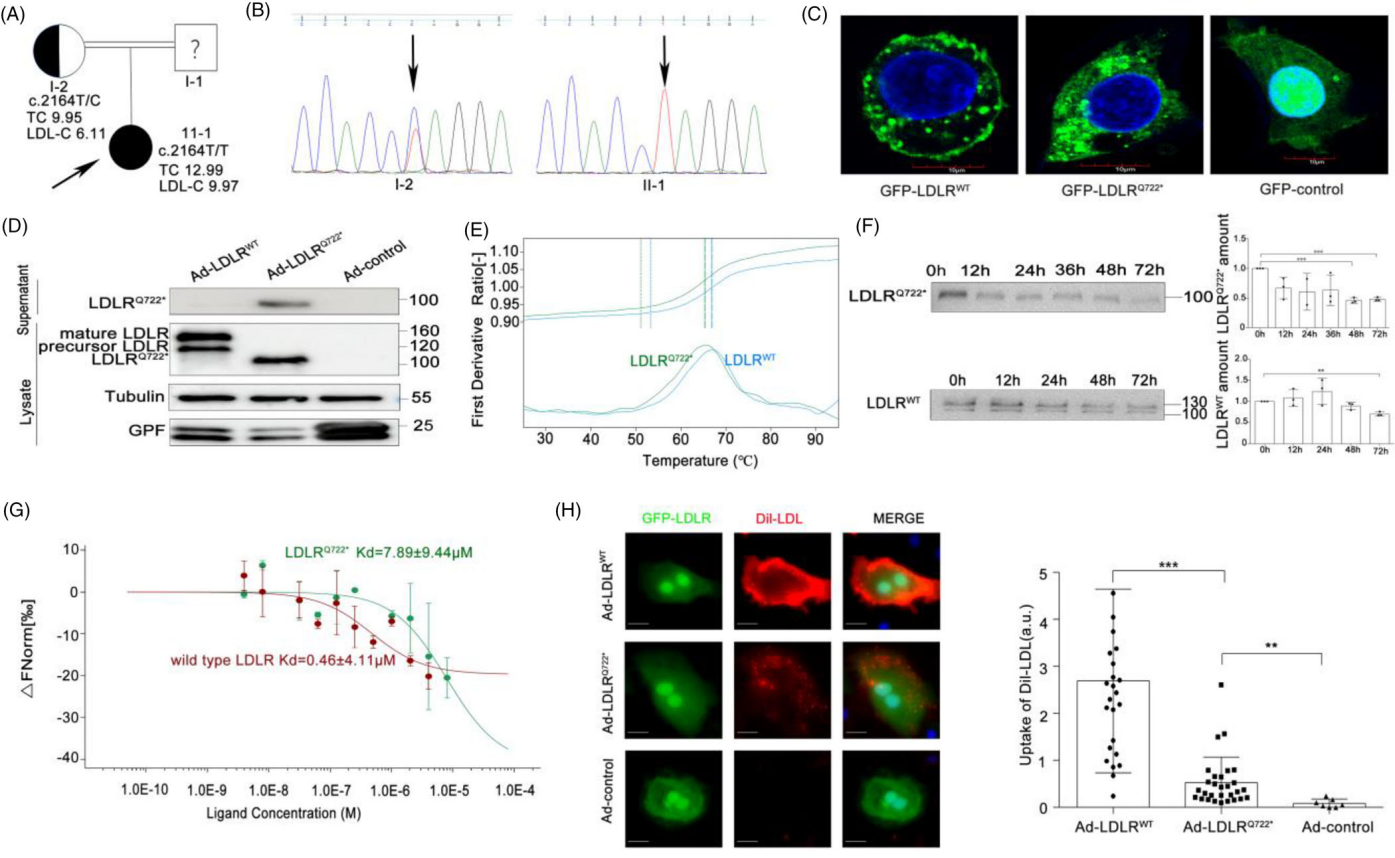
Discovery and functional validation of a novel truncated soluble LDLR^Q722*^. (A) Pedigrees of familial hypercholesterolemia (FH) family. Black arrow indicate proband and ? indicate unavailable for DNA analysis. (B) Sanger sequencing demonstrates c.C2164T mutation in the chromatogram from proband (II‐1) and heterozygous mother (I‐2). (C) HepG2 cells were infected with plasmids GFP‐LDLR^WT^, GFP‐LDLR^Q722*^, GFP‐control. LDLR subcellular localisation was visualised by confocal microscopy. LDLR (green), DAPI for nuclear (blue). Scale:10 μm. (D) *Ldlr^–/–^* primary hepatocytes were infected with recombinant adenovirus Ad‐LDLR^WT^, Ad‐LDLR^Q722*^ and Ad‐control. Western blot analysis of LDLR^Q722*^ in lysate and supernatant. Tubulin used as control. (E) Thermal stability was assessed by differential scanning fluorimetry (DSF) on wild‐type LDLR (*N*‐terminal residues 1–788, 1 ng/μl) and purified LDLR^Q722*^. Melt‐curve experiments starting at 25°C and with continuous 1% ramp to 95°C (roughly 1°C/min). (F) Wild‐type LDLR and LDLR^Q722*^ were placed at 37°C for 0–72 h and the protein amount was detected by the Western blot. (G) The binding affinities between purified LDLR^Q722*^(or wild‐type LDLR) and Dil labelled LDL were measured using microscale thermophoresis (MST), the ratio of detected fluorescence before and after the thermophoretic movement was plotted against the corresponding concentration. *Kd* model binding curves are depicted. Error bars represent standard error of three replicates (H) *Ldlr^–/–^* primary hepatocytes were infected with Ad‐LDLR^WT^, Ad‐LDLR^Q722*^, Ad‐control, then incubated with Dil‐LDL for 4 h, the uptake of Dil‐LDL was measured by confocal microscopy and normalised to green fluorescent protein (GFP) fluorescence intensity. GFP‐LDLR (green), 4',6‐diamidino‐2‐phenylindole (DAPI) for nuclear (blue), Dil‐LDL (red). Scale:10 μm. Data are expressed as mean ± SD. Statistical analyses, unpaired *t* test. * *p* < 0.05; ** *p* < 0.01; *** *p* < 0.001

Given the absence of the O‐linked sugar, transmembrane and intracellular domain of LDLR^Q722*^, LDLR^Q722*^ could not anchor to cell membrane (Figure 1C), secreted to extracellular and not glycosylated (Figure 1D, S1C‐E). The DSF showed that the thermostability of LDLR^Q722*^ (Tm = 65.3°C) seemed comparable to wild‐type LDLR (Tm = 66.9°C, Figure 1E), while the temporal stability of LDLR^Q722*^ (protein amount decreased by 54% for 48 h, *p *< 0.001) was poorer than wild‐type LDLR (decreased by 29.3% for 72 h, *p *< 0.001, Figure 1F). Although the affinity of LDL binding to LDLR^Q722*^ (*Kd *= 7.89±9.44 μM) was lower than its binding to wild‐type LDLR (*Kd *= 7.16±5.6 μM) (Figures 1G, S1F) by MST, LDLR^Q722*^ maintained the ability to combine with LDL due to the presence of intact LDL ligand binding domain. In line therewith, LDLR^Q722*^ increased the LDL uptake by 2.35‐fold (compared with control group, *p *= 0.0014), while decreased LDL uptake by 80.3% (compared with wild‐type LDLR, *p *< 0.001; Figures 1H, S1G) in *Ldlr^–/–^* primary hepatocytes. These results provide further evidence supporting the role of LDLR^Q722*^ in binding LDL and clearing up the extracellular LDL.

Next, the potential involvement of small extracellular vesicle (sEV) involved in LDLR^Q722*^ secretion was investigated. The transmission electron microscopy (TEM, Figure 2A), nanoparticle tracking analysis (NTA, Figure 2B), expression of marker proteins (Figure 2C) and GICT (Figure 2A) indicated that LDLR^Q722*^ was attached to the surface of sEV (denoted as sEV^Ad‐LDLR‐Q722*^), while wild‐type LDLR was not detected in sEV. The MST showed that sEV^Ad‐LDLR‐Q722*^ was able to bind LDL with a *Kd* of 0.012±2.91 μM (Figure 2D, S2A). *Ldlr^–/–^* primary hepatocytes were able to uptake sEV with a high efficiency (∼84%) (Figure 2F), meanwhile, the uptake of LDL in the sEV^Ad‐LDLR‐Q722*^ group was higher than that in the sEV^Ad‐LDLR‐WT^ (3.13‐fold, *p* < 0.001) and sEV^Ad‐control^ groups (2.68‐fold, *p* < 0.001) (Figure 2G). LDL also colocalised intracellularly with sEV^Ad‐LDLR‐Q722*^ (Figure 2E). These results demonstrated that sEV^Ad‐LDLR‐Q722*^are able to bind LDL and carried LDL into cells. Heparan sulphate proteoglycans (HSPG)^6^, ^7^ and clathrin‐mediated endocytosis^8^ play roles in the exosome transport. Competition assays were performed by adding exogenous heparin (HSPG inhibitors) to inhibit uptake of sEV^Ad‐LDLR‐Q722*^. Uptake of LDLR^Q722*^ and sEV was reduced by 66.8% (*p *= 0.003) (Figures 2H, S2B) and 37.1% (*p *= 0.002) (Figure 2J), respectively. Heparinase I, which cleaves and removes cell surface HSPG reduced uptake of LDLR^Q722*^ and sEV by 54.8% (*p *< 0.001) (Figures 2I, S2C) and 31.6% (*p *= 0.011) (Figure 2J), respectively. These results suggested that HSPG is a cell membrane bound receptor for sEV^Ad‐LDLR‐Q722*^. Chlorpromazine hydrochloride (CPZ, clathrin inhibitor) reduced LDLR^Q722*^ and sEV uptake by 41.82% (*p *= 0.002) (Figures 2K, S2H) and 26.3% (*p *= 0.018) (Figure 2L) (Figures S2D–G, S2I), respectively. Moreover, the clathrin on the cell membrane decreased by 71.98% (*p *< 0.001) (Figure 2M) following incubation with heparinase I, and LDLR^Q722*^ co‐located with clathrin Pearson correlation coefficient (PCC = 0.8194, Figure S2J) in cytoplasm of hepatocyte, supported the results that sEV^LDLRQ722*^ enter into cells via clathrin‐mediated endocytosis. Subsequently, sEV ^Ad‐LDLR‐Q722*^ were found to colocalise with EEA1 (a marker of early and intermediate endosome, Figure 3A), suggesting that sEV are delivered to early endosomes after internalisation. The acidic environment of the early endosome decreased the affinity between sEV^Ad‐LDLR‐Q722*^ and LDL from 4.98 μM to 0.013 μM when the pH was changed from 7.4 to 5.5 (Figure 3B). Then, LDLR^Q722*^ were released and secreted to the extracellular (Figure 3C), while LDL were trafficking to the lysosome for its degradation.

**FIGURE 2:**
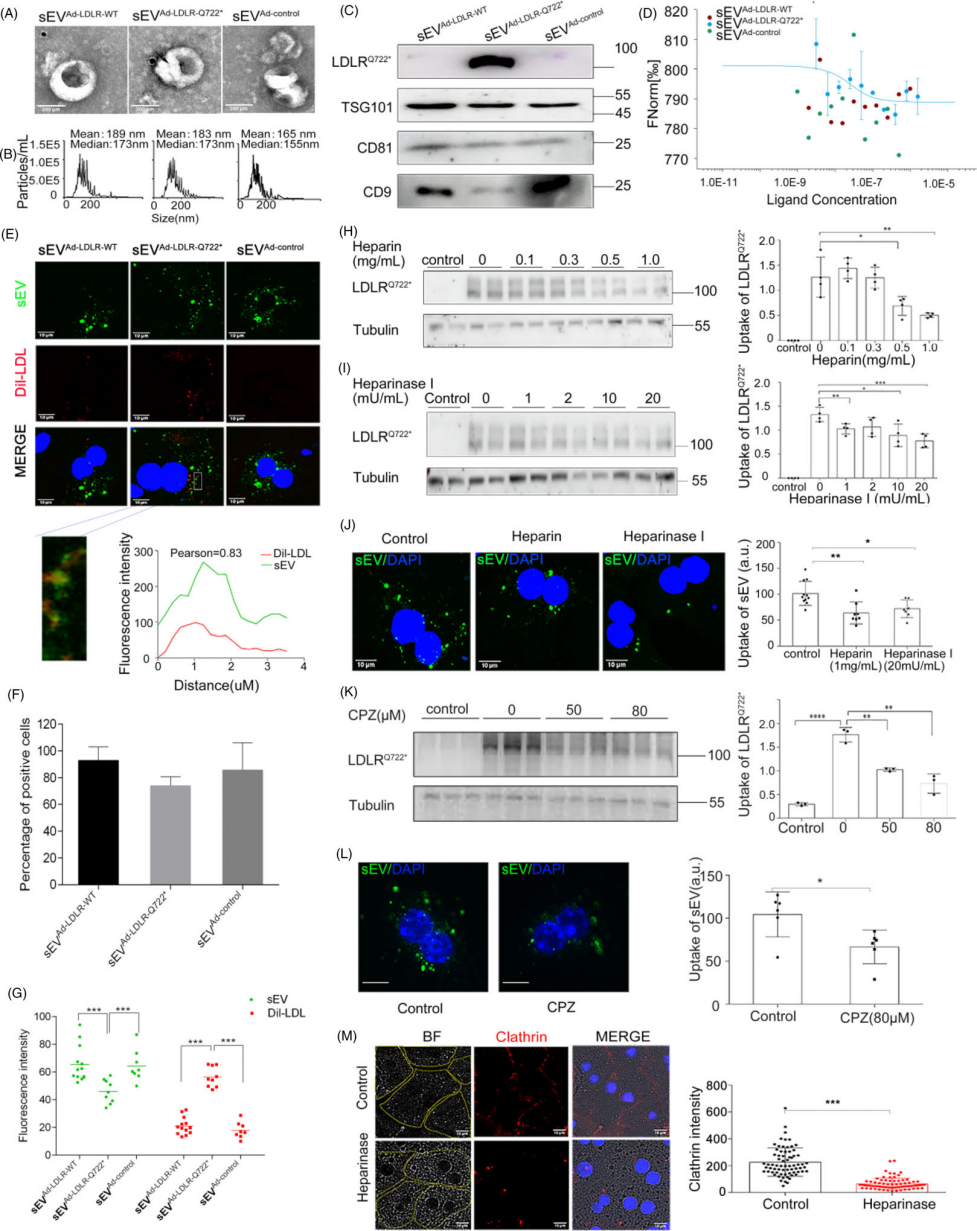
Heparan sulphate proteoglycans and clathrin mediated the endocytosis of small extracellular vesicles containing LDLR^Q722*^. (A) Transmission electron microscopy (TEM) image of sEV which isolated from the supernatant of HepG2 infected with Ad‐LDLR^WT^, Ad‐LDLR^Q722*^ and Ad‐control. Arrowheads indicate 5 nm gold particles after immunogold‐labelled with LDLR antibodies. Scale: 200 nm. (B) Nanoparticle tracking analysis (NTA) of sEV. (C) Western blot analysis of sEV marker proteins CD9, CD81, TSG101 and LDLR^Q722*^. (D) The binding affinities between sEV and Dil‐LDL were measured using MST. *Kd* model binding curves are depicted. (E) *Ldlr* ^–/–^ primary hepatocytes were cultured with Dil‐LDL and PKH67‐labeled sEV. Colocalisation of Dil‐LDL and PKH67‐sEV by confocal microscopy. Pearson correlation coefficient (PCC) was employed to quantify colocalisation. sEV(green), DAPI for nuclear (blue), Dil‐LDL (red). Scale: 10 μm. (F) Histograms showing the percent of uptake positive cells/all cells. (G) Intracellular fluorescence intensity of sEV and Dil‐LDL analysed by confocal microscopy. (H) *Ldlr* ^–/–^ primary hepatocytes were incubated without (control) or with Supernatants^Ad‐LDLR‐Q722*^ for 2 h in the presence of heparin, LDLR^Q722*^ uptake analysed by the Western Blot. (I) Same experiments as in (H) in the presence of heparinase I. (J) *Ldlr* ^–/–^ primary hepatocytes were incubated with PKH67‐sEV (30 μg/ml) for 4 h in the absence (control) or in the presence of heparin and heparinase I, sEV uptake analysed by confocal microscope. sEV (green), DAPI for nuclear (blue). Scale: 10 μm. (K) Same experiments as in (H) in the presence of chlorpromazine (CPZ), LDLR^Q722*^ uptake analysed by the Western blot. (L) Same experiments as in (H) in the presence of CPZ, sEV uptake analysed by confocal microscope. (M) Same experiments as in (H) and clathrin analysed by confocal microscopy. BF respected bright‐field, yellow line for cell contours, DAPI for nuclear (blue), CY3 for clathrin (red). Scale: 10 μm. Data are expressed as mean ± SD. Statistical analyses, unpaired *t* test. * *p* < 0.05; ** *p* < 0.01; *** *p* < 0.001

**FIGURE 3:**
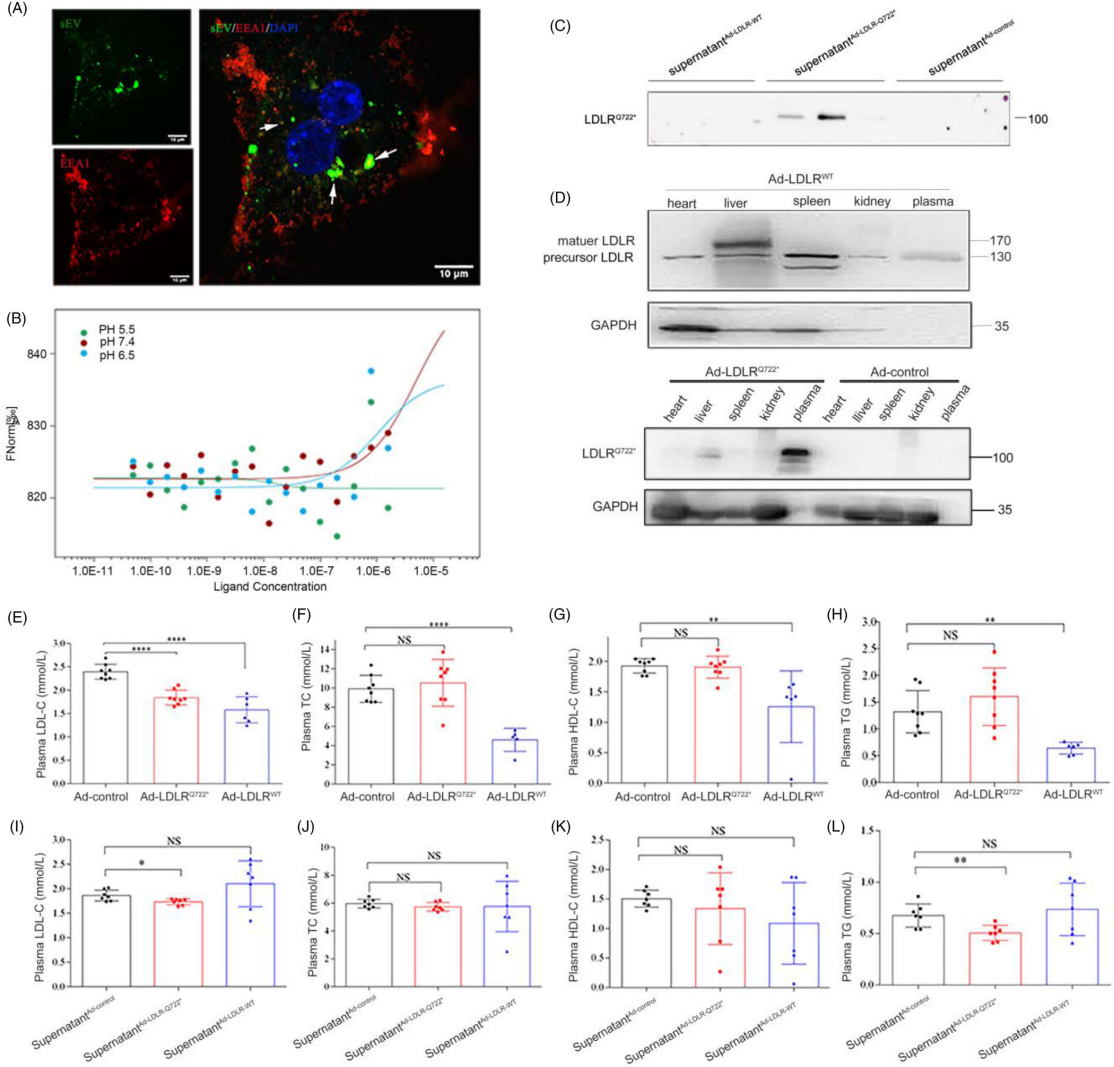
LDLR^Q722*^ reduced plasma LDL‐C levels in *Ldlr* ^–/‐^ mice. (A) *Ldlr* ^–/–^ primary hepatocytes were incubated with PKH67 labelled sEV for 2 h, colocalisation of sEV and EEA1(a marker of early and intermediate endosome) analysed by confocal microscopy. DAPI for nuclear (blue), CY3 for EEA1 (red). Arrows show example of localisation between sEV and EEA1. Scale: 10 μm. (B) The binding affinities between sEV^Ad‐LDLR‐Q722*^ and Dil‐LDL were measured using MST. *Kd* model binding curves are depicted. (C) Ad‐LDLR^WT^, Ad‐LDLR^Q722*^ and Ad‐control were transfected into HepG2 cell, the supernatant^Ad‐LDLR‐WT^, supernatant^Ad‐LDLR‐Q722*^, supernatant^Ad‐LDLR‐control^ were collected. *Ldlr* ^–/–^ primary hepatocytes were incubated in supernatant supplemented with LDL for 2 h, then replace fresh serum‐free medium, analysed for LDLR^Q722*^ secretion in the fresh serum‐free medium after 2 h by the Western blot. (D) *Ldlr* ^–/–^mice were injected with 200 μl, 5×10^11^ vp/ml Ad‐LDLR^WT^ (*n* = 6), Ad‐LDLR^Q722*^ (*n* = 8) and Ad‐control (*n* = 8) via tail vein for 2 weeks. Expression of LDLR^Q722*^ in different tissues by the Western blot. (E–H) Plasma LDL‐C, TC, HDL‐C, TG were determined by automatic biochemical analyser. (I–L) *Ldlr* ^/–^ mice were injected with 200 μl supernatant ^Ad‐control^ (*n* = 7), supernatant^Ad‐LDLR‐Q722*^ (*n* = 7) and supernatant ^Ad‐LDLR‐WT^ (*n* = 7) every other day via tail vein for 2 weeks, plasma LDL‐C, TC, HDL‐C and TG were determined. Data are expressed as mean ± SD. Statistical analyses, unpaired *t* test. * *p* < 0.05; ** *p *< 0.01; **** *p *< 0.0001; NS: not statistically significant

In vivo, mice were injected with recombinant adenovirus, LDLR^Q722*^ was highly expressed in the plasma (Figure 3D). Adenovirus‐mediated LDLR^Q722*^ decreased plasma LDL‐C by 23.14% (2.40 to1.84 mmol/L, *p *< 0.001), and no significant change in TC, TG and HDL‐C (Figure 3E–H, S3). sEV‐mediated LDLR^Q722*^ directly decreased plasma LDL‐C and TG decreased by 8% (*p *= 0.01) and 24.93% (*p *= 0.006), respectively, and no significant differences were observed in the TC and HDL‐C (Figure 3I–L). Although the lipid‐lowering effect of the adenovirus‐mediated wild‐type LDLR was better than that of the LDLR^Q722*^, its high immunogenicity and short‐term expression limits clinical application.^9^ LDLR^Q722*^ attached to the surface of sEV (instead of encapsulating LDLR mRNA or DNA inside sEV^10^), as “natural nanoparticles” was safer and easier to use. It is thus promising to develop an sEV‐based LDLR‐protein delivery strategy for the treatment of FH.

In conclusion, we identified a novel homozygous pathogenic mutation *LDLR* c.C2164T (p. Q722*) that caused FH in Chinese, and no other potential pathogenic mutation in any other screened genes (Table S3). The mutation formed a novel truncated soluble LDLR^Q722*^. LDLR^Q722*^ was secreted via the sEV. LDLR^Q722*^ located in sEV was able to bind to LDL, and subsequently entered cells via cell‐surface heparan sulphate proteoglycans (HSPG) and clathrin‐mediated endocytosis. Thus, cleared circulating LDL and reduced plasma LDL‐C level (Figure 4). This study provided new insights into the genetic diagnosis and treatments of FH.

**FIGURE 4:**
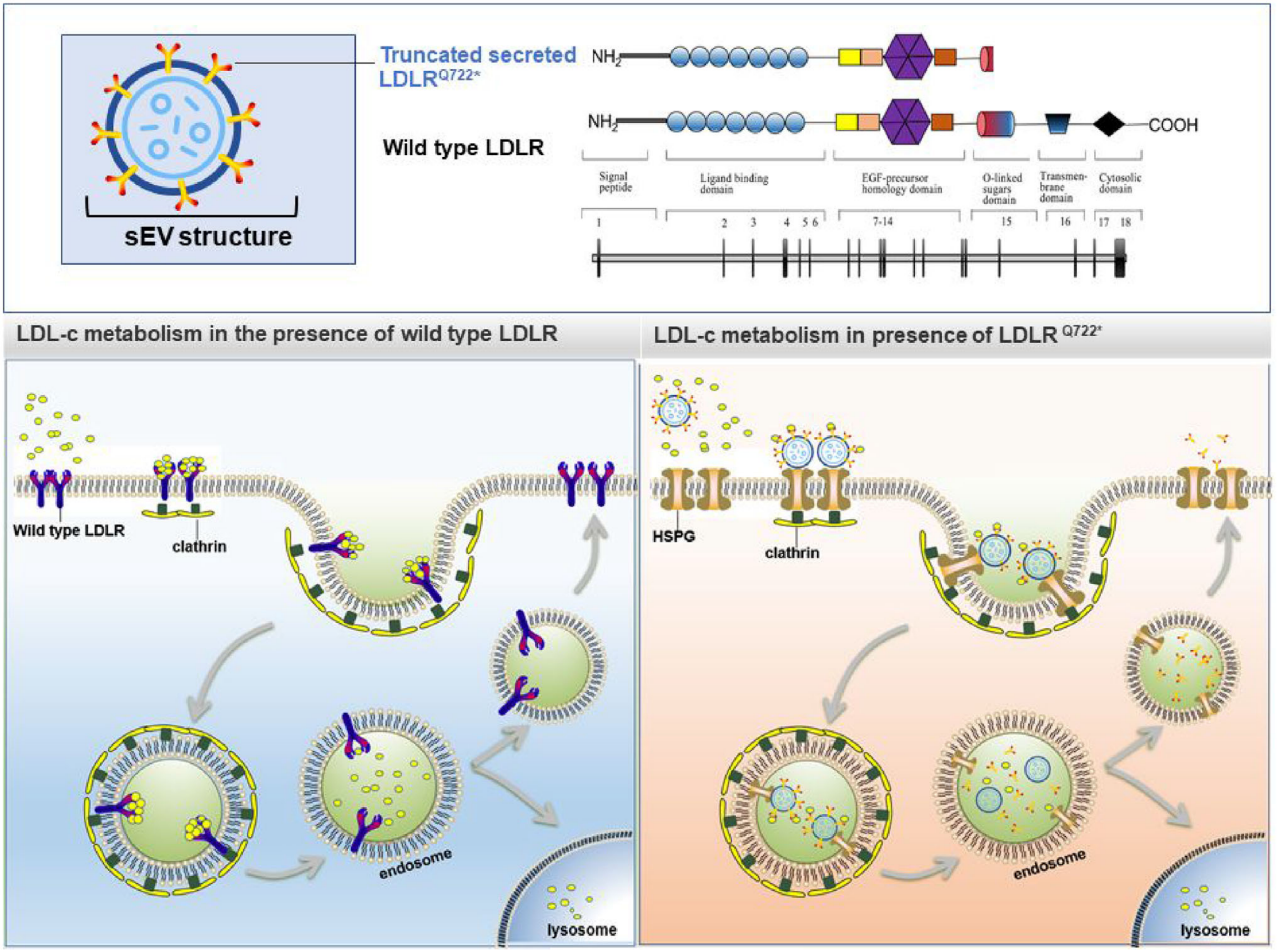
Upper graph: the c.C2164T mutation produced a truncated soluble protein LDLR^Q722*^ compared with wild‐type LDLR. LDLR^Q722*^ was secreted and attached to the surface of the small extracellular vesicle (sEV). Lower right graph: in the presence of LDLR^Q722*^, LDLR^Q722*^ carried by sEV binds to LDL, subsequently enters cells via heparan sulphate proteoglycans (HSPG) and clathrin‐mediated endocytosis and transports to early endosome, in which the sEV^Ad‐LDLR‐Q722*^/LDL complex was dissociated. Then, LDLR^Q722*^ were released and secreted to the extracellular, while LDL trafficking to the lysosome for its degradation. Lower left graph: in the presence of wild‐type LDLR, LDL binds to the cell surface LDLR and the LDLR*–*LDL complex is internalised via clathrin‐mediated endocytosis, followed by lysosomal degradation of LDL, but the LDLR is recycled on the cell surface

## CONFLICT OF INTEREST

The authors declare that there is no conflict of interest that could be perceived as prejudicing the impartiality of the research reported.

## Supporting information

Supporting information
