# Supplementary material for: Small extracellular vesicles containing LDLRQ722* protein reconstructed the lipid metabolism via heparan sulphate proteoglycans and clathrin‐mediated endocytosis
Source: Clin Transl Med. 2022 Mar 28;12(3):e773. doi: 10.1002/ctm2.773 (PMC8958347; doi:10.1002/ctm2.773)
Supplement: Supplementary file 1 — Supporting information [file CTM2-12-e773-s001.pdf]

Supplementary Information

Small extracellular vesicles containing LDLR^{Q722*} protein reconstructed the lipid metabolism via heparan sulfate proteoglycans and clathrin-mediated endocytosis

Yingchao Zhou,[1,2,†] Qiang Xie,[3,†] Silin Pan,[2,†]; Jianfei Wu,[1] Xiangyi Wang,[1] Zhubing Cao,[1] Mengru Wang,[1] Lingfeng Zha,[4] Mengchen Zhou,[4] Qianqian Li,[1] Qing Wang,[1] Xiang Cheng,[4] Gang Wu,[5] Xin Tu,[1,*]

[1] Key Laboratory of Molecular Biophysics of the Ministry of Education, College of Life Science and Technology, Center for Human Genome Research, Cardio-X Institute, Huazhong University of Science and Technology, Wuhan, China.

[2] Heart Center, Women and Children's Hospital, Qingdao University, Qingdao, China.

[3] Department of Cardiology, The First Affiliated Hospital of Xiamen University, Xiamen, China.

[4] Department of Cardiology, Union Hospital, Tongji Medical College, Huazhong University of Science and Technology, Wuhan, China

[5] Department of Cardiology, Renmin Hospital of Wuhan University, Wuhan, China.

[†] These authors contributed equally to this work.

*Correspondence: Xin Tu at Key Laboratory of Molecular Biophysics of Ministry of Education, College of Life Science and Technology, Center for Human Genome Research, Cardio-X Institute, Huazhong University of Science and Technology, Luoyu road 1037, Wuhan, 430074, China. E-mail: xtu@hust.edu.cn

Supplementary results

Pathogenic mutation *LDLR* c.C2164T was identified in a Chinese FH family

Whole-exome sequencing assay identified 52,559 (I-2) and 51,603 (II-1) variations, respectively. The data was analyzed following the generally-accepted standard process[14]: Assuming that no causal mutation for FH or a rare variant was reported in the known database, we excluded variants with global MAF>0.01 in the dbSNP138 or 1000 Genomes Project database, 2,345 (I-2) and 2,953 (II-1) variations were reserved. Among these variants, only 1,989 (I-2) and 1,178 (II-1) variations were loss-of-function mutations. Considering that the causal mutation is inherited in the dominant mode, we hypothesized that the mutant gene in II-1 is a homozygote whereas in the I-2 is a heterozygote. In this way, only 23 variants were left. To further confirm the mutant in this FH family, we performed sanger sequencing, identifying three variants located at *CLEC14A*, *CDKL5*, and *LDLR*. However, the mutations in *CLEC14A* and *CDKL5* were predicted to be benign by SIFT and PolyPhen-2, respectively. Only the variant at LDLR was left in the candidate list. Finally, we

identified *LDLR* c.C2164T (Figure 1B) is the causal mutation of the Chinese FH patient.

The homozygote nonsense mutation c.C2164T (CCDS12254、NM_000527.4) was localized in exon 15 (coding O-linked sugar domain) of *LDLR* which turns CAG into TAG, leading to early termination of translation, translated into a truncated protein (containing 1-722 amino acids), which lost the transmembrane and intracellular domains of LDLR (Supplementary material, Figure S1A). Alignment of the mutation c.C2164T in different species, as indicated Supplementary material online, Figure S1B, the mutated sites are highly conserved. According to the latest ACMG genetic variation classification criteria and guidelines, *LDLR* c.C2164T could be classified as pathogenic (PVS1).

Since LDLR, PCSK9 and APOB are reported as the main pathogenic genes of FH (also recommended by the 2019 ESC/EAS Guidelines for FH patients), the three genes were analyzed particularly. All variants of the three genes were summarized in Table S3. We chose to focus on the c.1056C>T (in LDLR), c.277C>T (in PCSK9) and c.4265A>G (in APOB) since the MAF of these variants <0.01. However, these three variants were reported as benign or Likely benign. Therefore, we identified that LDLR c.C2164T is the causal mutation in this Chinese FH patient.

Supplementary Methods

Clinical characteristics

The proband was a female aged 32 years with a previous diagnoses of coronary heart disease and hyperlipidemia. The plasma lipid profile described below: LDL-C 9.97mmol/L, TC 12.99mmol/L, HDL-C 1.91mmol/L, TG 1.38mmol/L. According to the description from proband, there is no family history of FH and cardiac diseases in the family (The proband's mother plasma LDL-C 6.11mmol/L, TC 9.95mmol/L, HDL-C 1.18mmol/L, TG 1.48mmol/L and no history of coronary heart disease. The proband's father died from cancer many years ago, grandparents have no a definite history of hyperlipidemia and coronary heart disease). The present study was approved by the Ethics Committee of Huazhong University of Science and Technology. The study adhered to the guidelines of the Declaration of Helsinki. Written Informed Consent was obtained from each participant.

Whole-exome sequencing and variant calling

Whole-exome sequencing of proband and her mother were performed by a Next-Generation Sequencing (NGS) strategy to detect the causal genes of the FH pedigree. The variants were annotated with SeattleSeq Annotation 138 and sequentially filtered according to the following criteria: (1) Variants in the database for dbSNP 138 or 1000 Genomes Project with global minor allele frequency >0.01 were excluded; (2) Loss-of-function (missense, frameshift, and splicing) and transcription factor binding site variants were included; (3) Variants in proband is a homozygote while that of her mother is a heterozygote; (4) Variations were verified by Sanger sequencing.

Plasmids and transfections

The full-length coding region of wide type *LDLR* and the full-length c.C2164T site-directed mutagenesis mutant LDLR was amplified using human cDNA and subcloned into the pEGFP-C1vector. The constructs were named GFP-LDLR^{WT} and GFP-LDLR ^{Q722*}, respectively. HepG2 cells were transiently transfected via Lipofectamine 2000 (Invitrogen, USA) according to the manufacturer's instruction.

2.4 Generation of recombinant adenoviral vectors

The full-length coding region of wide type *LDLR* (2583bp, encoding 860 residue) and truncated segment (1-2164bp, encoding 1-722 residue) was subcloned into the pAV-CMV>EGFP adenoviral vectors. The construct was named Ad-LDLR^{WT} and Ad-LDLR^{Q722*} respectively. The connection sequence T2A (GGAAGCGGAGAGGG CAGGGGAAGTCTTCTAACATGCGGGGACGTGGAGGAAAATCCCGGCCCC) was added to the *C*-terminal of LDLR and the *N*-terminal of EGFP. T2A encodes a self-processing peptide[11], the translated polyprotein was cleaved into two proteins at the *C*-terminus of the T2A region. The products were an upstream LDLR fused with T2A and a complete downstream EGFP protein. The T2A link successfully prevented the interference of expression between the individual genes.

Cell culture

HepG2 cells were purchased from the American Type Culture Collection (Manassas, USA). Primary hepatocyte was isolated and from *Ldlr*$^{-/-}$ mice described by Primary Mouse Hepatocytes (http://www.mouselivercells.com/index.html). HepG2 were cultured in Dulbecco's modified Eagle's medium (DMEM) supplemented with

10% fetal bovine serum (FBS). Primary hepatocytes were cultured in hepatocyte medium supplemented with 5% FBS, 1% hepatocyte growth supplement (sciencell, USA; Cat.5252) and 1% penicillin/streptomycin solution (sciencell, USA; Cat.0503). Cells were cultured in a humidified incubator at 37 °C under a 5% CO_2 atmosphere.

Western bolt

Whole cell lysates or the supernatants were separated with SDS–PAGE and transferred onto polyvinylidene fluoride (PVDF) membranes. The blots were blocked with 5% nonfat dry milk at room temperature for 2 h, incubated overnight at 4°C with the corresponding primary antibodies and incubated with HRP-conjugated secondary antibodies at room temperature for 2 h. The signals were developed in SuperSignal ELSA Femto Maximum Sensitivity Substrate (Thermo Fisher Scientific, USA). ChemiDoc XRS (Bio-Rad Laboratories, USA) was used to detect the signals. Quantity One Basic v. 4.4.0 (Bio-Rad Laboratories, USA) quantified the band intensities.

Immunofluorescence

The cells climbing slice were fixed with 4% paraformaldehyde at room temperature for 30 min and washed thrice with PBS for 10 min. The cells were permeabilized for 15 min with 0.2% Triton X-100. Next, cells were blocked for 2 h with PBS-5% BSA, then incubated for another 12-14h in corresponding primary antibodies. They were subjected to three washings with PBS and incubated at room temperature with fluorescent secondary antibodies for 2h. After washing with PBS, the cells were observed under a confocal microscope (Olympus FV1000, USA).

Ligand binding blot

HepG2 cells transfected with Ad-$LDLR^{WT}$, Ad-$LDLR^{Q722*}$ and Ad-control for 48 h, the supernatant was denoted by $supernatant^{Ad\text{-}LDLR\text{-}WT}$, $supernatant^{Ad\text{-}LDLR\text{-}Q722*}$, and $supernatant^{Ad\text{-}control}$ separately. The supernatant spotted on nitrocellulose membrane, blots were preblocking for 1h in TBSM (TBS containing 5% delipidated milk) and then incubated for 2h with the LDL (30μg/mL). The membranes were immunoblotted with LDLR (Proteintech, No.10785-1-AP;) and APOB (Proteintech, No.20578-1-AP;) antibody at 4°C for 16h followed by western blotting.

Protein stability assay

Commercial wide type LDLR protein (N-terminal residues 1-788, Cat: 10231-H08H) was purchased from Sino Biological. $LDLR^{Q722*}$ were purified from $supernatant^{Ad\text{-}LDLR\text{-}Q722*}$ (HepG2 cells transfected with Ad-$LDLR^{Q722*}$) as described[12]. $LDLR^{Q722*}$ were analyzed by SDS-PAGE and identification by mass spectrometry (ekspertTMnanoLC; AB Sciex TripleTOF 5600-plus). The thermo stability of $LDLR^{Q722*}$ and wide type LDLR protein was performed using the NanoDSF (Nano Temper Prometheus NT.48, German) according to the manufacturer's instructions. DSF is sensitive to the tryptophan exposure as protein unfolds. Temperature ramping was run from 25°C to 95°C at 1°C increment per minute. The $supernatant^{Ad\text{-}LDLR\text{-}Q722*}$ and the wildtype LDLR were placed at 37°C for 0-72h. Western blotting was performed to protein stability over time.

Binding affinity assay

The binding affinities between Dil (1,1′-Dioctadecyl-3,3,3′,3′-tetramethylindocarb-ocyanine perchlorate) labeled LDL and purified $LDLR^{Q722*}$ were measured using microscale thermophoresis (MST). Dil-LDL (50nM) and purified $LDLR^{Q722*}$ (2.5μM, gradient dilution) were mixed in PBS, then the samples were loaded into silica capillaries and temperature-induced fluorescence changes were measured on a Monolith™ (NanoTemper) at 25 °C by using 20% LED-power and 40% MST-power. The same test condition was applied to detected the binding affinities between Dil-LDL and wide type LDLR protein. The Kd fit model describes a molecular interaction with a 1:1 stoichiometry according to the law of mass action. Kd fit function of Mo.Affinity Analysis v2.3 was used to fit curve and calculate the value of dissociation constant (Kd).

CCK8 cell viability assay

Cell suspension were placed in a 96-well plate that had been pre-incubated for 24 h. Then 10, 20, 30, 50, 80, or 100μM chlorpromazine hydrochloride (CPZ; Sigma, USA, T8158) was added to the plate with six repetitions per concentration. After 2 h, 10μL CCK8 solution was added to each well. The plates were incubated for 2 h at 37°C and absorbances were measured in a microplate reader (MultiSkan, Thermo Fisher Scientific,USA) at 450 nm.

Small extracellular vesicles purification and fluorescence labeling

HepG2 cells were transfected with Ad-LDLR^{WT}, Ad-LDLR^{Q722*}, Ad-control and cultured in serum-free medium for 48h. Exosomes were isolated from culture medium of transfected cells using ultracentrifugation method: (1) The supernatants were centrifuged at 2,000×*g* 4°C for 20 min to remove dead cells; (2) The cell debris was removed after centrifuged at 10,000×*g* 4℃ for 30 min; (4) The supernatants were recentrifuged at 100,000×*g* 4°C for 70 min; (5) The pelleted exosomes were resuspended in 1×PBS and centrifuged at 100,000×*g* 4°C for 70 min; (6) The purified exosomes was resuspended in 1×PBS.

The sEV were subjected to PKH67 (Umibio, UR52121,) fluorescent labeling according to the manufacturer's recommendations and protein content was determined by using the BCA protein assay before further analyses.

sEV and Dil-LDL internalization assays

Cells were pretreated with inhibitors (CPZ 0-80μM; Heparin 0.1-1mg/mL; Heparinase 0.001-0.02U/mL) for 1 h, then incubated with PKH67-labeled EVs (30μg/mL) and Dil-LDL (30μg/mL) for 4 h in the presence of inhibitors. Subsequently, cells were washed with PBS and fixated in 4% paraformaldehyde. Finally, the cell climbing slices were added fluorescence-quenching agent (containing DAPI) for sealing. The sEV and Dil-LDL internalization were observed under confocal microscope (Olympus FV1000, USA).

In vivo study

All experiments were performed in accordance with the Guide for the Care and Use of Laboratory Animals published by the US National Institutes of Health [NIH Publication No.8, revised 2011]. All procedures were approved by the Animal Use Subcommittee at the Huazhong University of Science and Technology, P. R. China.

C57BL/6J (male, 8-weeks) and homozygous *LDLR*-deficient mice (male, 8-weeks) were obtained from the Animal Model Research Center of Nanjing University (Nanjing, China). Starting at 12 weeks, the mice were placed on a standard diet and maintained on it throughout the experiments. The mice received 200μL of 5×10^{11}vp/mL Ad-$LDLR^{WT}$, Ad-$LDLR^{Q722*}$ and Ad-control vector via tail vein injection. Two weeks later, euthanasia was performed with an intraperitoneal injection of 240 mg/kg of pentobarbital sodium, followed by blood collection via cardiac puncture. The heart, liver, spleen and kidney tissue were taken to prepare a tissue homogenate. Plasma total cholesterol (TC), low-density lipoprotein cholesterol (LDL-C), high-density lipoprotein cholesterol (HDL-C), and triglyceride (TG) levels were determined by automatic biochemical analyzer (Chemray 240, CN) according to the manufacturer's instructions.

Statistical analysis

Data are means ±SD and were analyzed by a two-tailed Student's *t*-test. *p* values <0.05 were considered significant, and the statistical tests and sample size (*n* values) used in the experiments are specified in the figure legends. In the figures, asterisks

indicate the *P* values: *$p < 0.05$, **$p < 0.005$, and ***$p < 0.001$. GraphPad Prism 6.0b (GraphPad software, La Jolla, CA, USA) were used for the statistical analyses.

Supplementary Figures

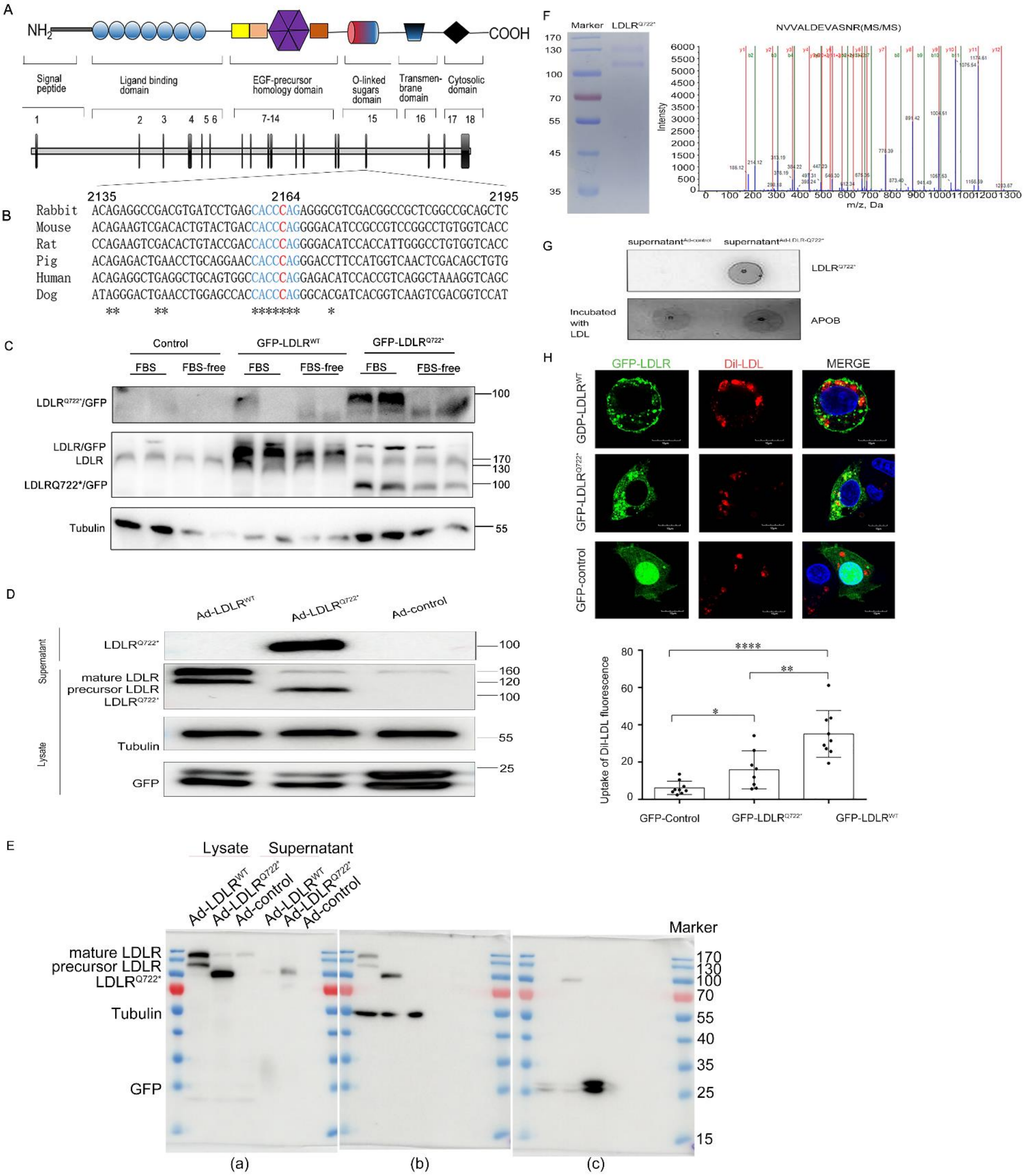

Figure S1. Discovery and functional validation of a novel truncated soluble LDLR^{Q722*}

(A) Schematic of the LDLR gene construct. (B) The conservation of the sequence is indicated for different species. Stars point out the consensus sequence, c.C2164T highlighted in red. (C) HepG2 cells were infected by overexpressed plasmids GFP-LDLR^{WT}, GFP-LDLR^{Q722*}, GFP-control, western blot analysis of mutant LDLR in lysate and supernatant. Tubulin as control. (D) HepG2 cell were infected with recombinant adenovirus Ad-LDLR^{WT}, Ad-LDLR^{Q722*} and Ad-control. Western blot analysis of LDLR^{Q722*} in lysate and supernatant. Tubulin used as control. (E) Recombinant adenovirus Ad-LDLRWT, Ad-LDLRQ722*, Ad-control infected HepG2 cells. Western blot analysis of LDLRQ722* in lysate and supernatant. (a) Uncropped Western blot were performed using LDLR antibody. (b)
The antibody was eluted and then incubated with Tubulin antibody. Tubulin was used as a loading control. (c) The antibody elution process was repeated once
and then incubated with GFP antibody. GFP were used as a control for transfection efficiency. (F) HepG2 cell were transfected with recombinant adenovirus Ad-LDLR^{Q722*} which expressing LDLR^{Q722*}, the supernatants containing LDLR^{Q722*} were analyzed by SDS-PAGE gel, the enriched protein bands were used for mass spectrum (left). Tandem mass-spectrometry (MS/MS) spectrum confirming the identity of LDLR (a peptide of 428NVVALDTEVASNR440) (right). (G) Supernatant$^{Ad\text{-}control}$ and supernatant$^{Ad\text{-}LDLR\text{-}Q722*}$ were spotted on the nitrocellulose membrane and incubated with LDL, ligand binding blot analysis of APOB (LDL marker) immunoblots were used to observe the ability of LDLR^{Q722*} combine with LDL. (H) HepG2 cells were infected by plasmids GFP-LDLR^{WT}, GFP-LDLR^{Q722*}, GFP-control and incubated with Dil-LDL. The Dil-LDL uptake was visualized by confocal microscopy and normalized to GFP fluorescence intensity. Dil-LDL (red), LDLR (green), DAPI for nuclear (blue), scale:10μm. Data were expressed as mean ± SD. Statistical analyses, unpaired t test. $*p < 0.05$; $** p < 0.01$; $**** p < 0.0001$.

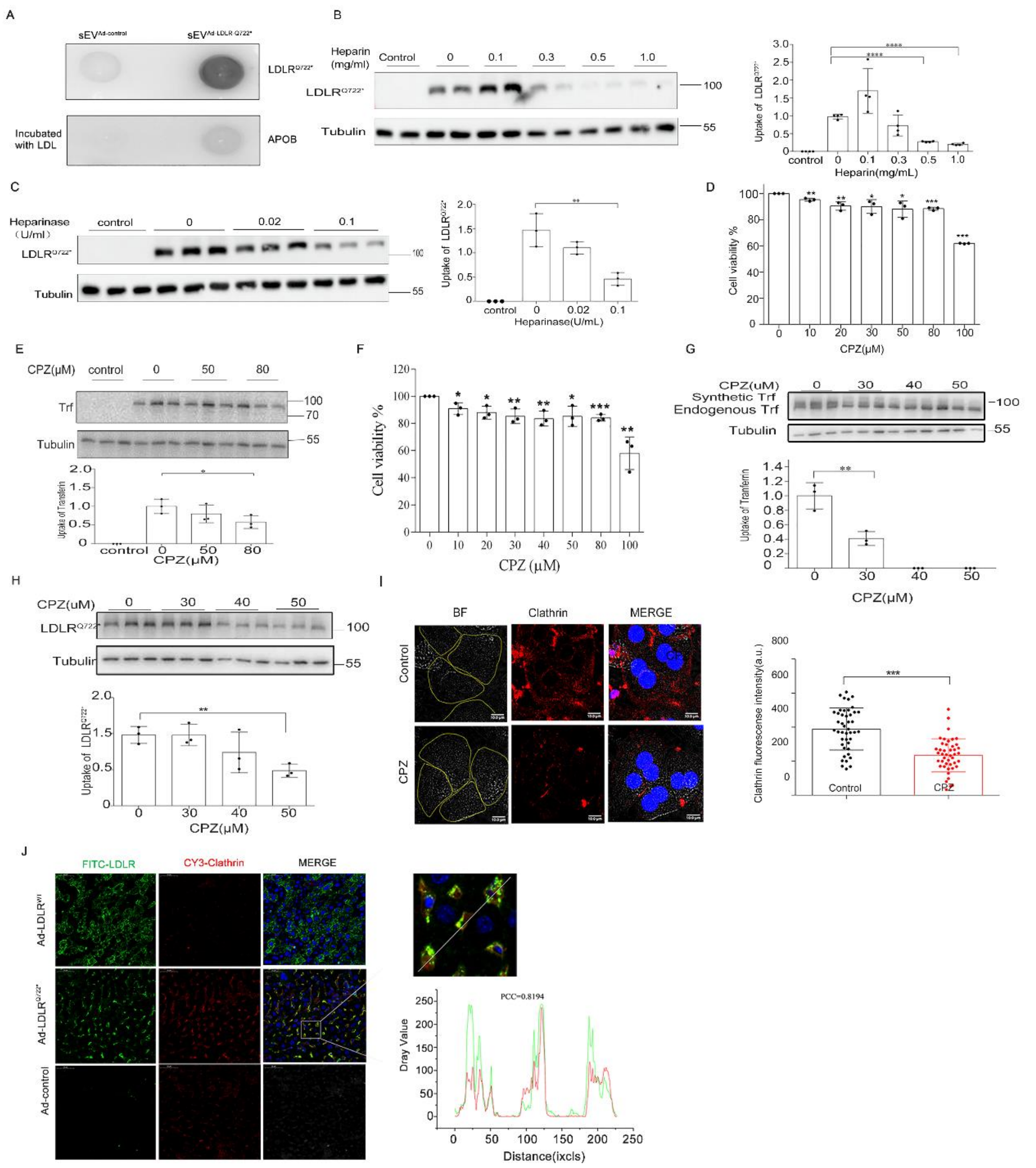

Figure S2. Heparan sulfate proteoglycans and clathrin mediated the endocytosis of small extracellular vesicles containing LDLR^{Q722*}

(A) sEV$^{Ad\text{-}control}$ and sEV$^{Ad\text{-}LDLR\text{-}Q722*}$ isolated from the supernatant of HepG2 infected with Ad-control and Ad-LDLR^{Q722*}. sEV were sampled on nitrocellulose membrane and incubated with LDL for 2h. APOB (LDL marker) immunoblots were used to

detect the ability of $LDLR^{Q722*}$ bond to LDL. (B) HepG2 cells were incubated without (control) or with $supernatant^{Ad\text{-}LDLR\text{-}Q722*}$ for 2 h in the presence of heparin, $LDLR^{Q722*}$ uptake analyzed by western blot. (C) Same experiments as in (B) in the presence of heparinase I. $LDLR^{Q722*}$ uptake analyzed by western blot. (D) CCK-8 detected the cell viability of *Ldlr*$^{-/-}$ primary hepatocytes incubated with chlorpromazine hydrochloride (CPZ, a calthrin inhibitor). (E) *Ldlr*$^{-/-}$ primary hepatocytes were incubated with transferrin for 2 h in the presence of CPZ, then analyzed for transferrin uptake (as a control to measure clathrin activity) by western blot. (F) CCK-8 detected the cell viability of HepG2 cells. (G) Same experiments as in (E) in HepG2 cells. (H) Same experiments as in (E) in HepG2 cells, $LDLR^{Q722*}$ uptake analyzed by western blot. (I) *Ldlr*$^{-/-}$ primary hepatocytes were incubated with $Supernatants^{Ad\text{-}LDLR\text{-}Q722*}$ for 2 h in the absence (control) or in the presence of 0.02U/mL heparinase I, clathrin analyzed by confocal microscopy. BF respected bright-field, yellow line for cell contours, DAPI for nuclear (blue), CY3 for clathrin (red). Scale: 10μm. (J) *Ldlr*$^{-/-}$ mice were injected with Ad-$LDLR^{WT}$ (n=6), Ad-$LDLR^{Q722*}$ (n=8) and Ad-control (n=8), the liver tissue was collected for immunofluorescence staining. DAPI for nuclear (blue), CY3 for clathrin (red), LDLR (green). Scale: 50μm. Data are expressed as mean ± SD. Statistical analyses, unpaired t test. * $p < 0.05$; ** $p < 0.01$; *** $p < 0.001$; **** $p < 0.0001$.

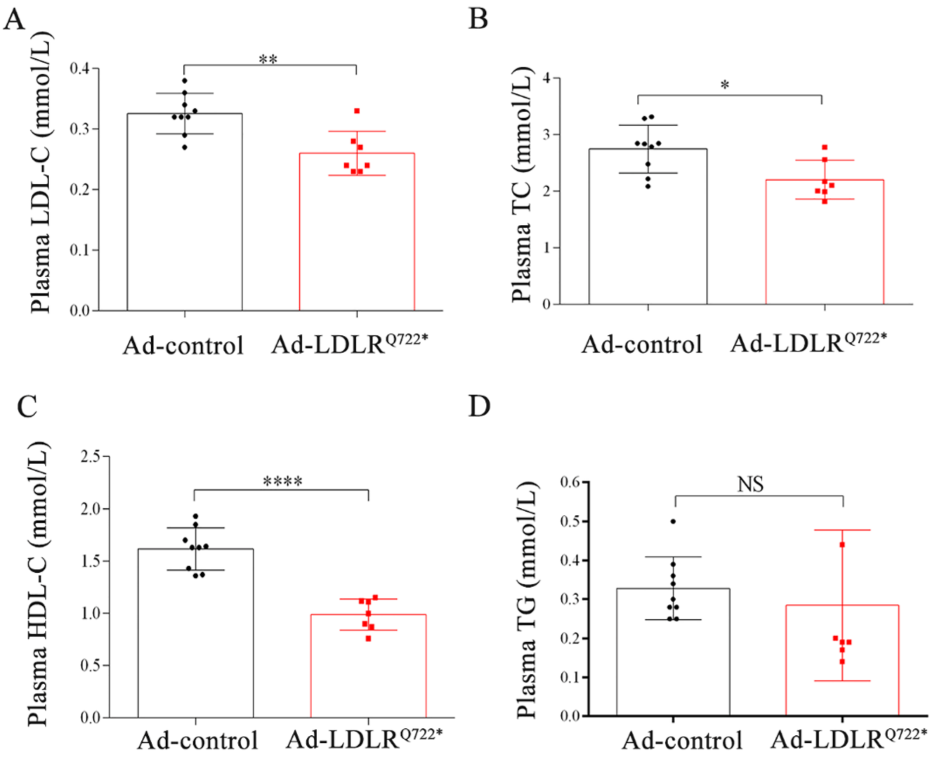

Fig S3 LDLR^{Q722*} reduced plasma LDL-C levels in C57BL/6J mice

(A-D) C57BL/6J mice were injected with 200 μL, 5×10^{11}vp/mL Ad-LDLR^{WT} (male, 12-week, n=9), Ad-LDLR^{Q722*} (male, 12-week, n=7) via tail vein for 2 weeks. Plasma LDL-C, TC, HDL-C and TG were determined by automatic biochemical analyzer. Data are expressed as mean ± SD. Statistical analyses, unpaired t test. * $p < 0.05$; ** $p < 0.01$; NS, not statistically significant.

Supplementary Tables

Table S1. Deletion and frameshift deletion homozygous mutation in LDLR gene

Location	DNA change (cDNA)	Protein effect	Frequency in ExAC
E1	c.11G>A	p.Trp4*	N
E2	c.97C>T	p.Gln33*	0.0001651
E3	c.272delG	p.Gly91Alafs*114	N
E4	c.337dupG	p.Glu113Glyfs*17	N
E4	c.337G>T	p.Glu113*	N
E4	c.682G>T	p.Glu228*	0.0001709
E6	c.892delA	p.Met277Trpfs*72	N
E6	c.916_919dupTCAG	p.Asp307Vals*3	N
E6	c.971delG	p.Gly303Alafs*46	N
E7	c.980_981insA	p.His327fs*5	N
E7	c.1045delC	p.Gln349Serfs*21	N
E7	c.1048C>T	p.Arg350*	0.000008272
E8	c.1176C>A	p.Cys392*	0.000008354
E8	c.1070delA	p.Glu357Glyfs*13	N
E8	c.1178delA	p.Lys393Argfs*20	N
E9	c.1342C>T	p.Gln448*	N
E13	c.1878delA	p.Ala627Profs*38	N
E14	c.2043C>A	p.Cys681*	0.000008255
E14	c.2054delC	p.Pro685Argfs*24	N

E15	c.2207delG	p.Gly676Alafs*33	N
E15	c.2264_2273delCCACCCCTGG	p.Ala755Glyfs*7	N
E15	c.2270delC	p.Pro757Leufs*8	N
E15	c.2271delT	p.Leu759Serfs*6	N
E15	c.2286delG	p.Glu763Argfs*2	N
E16	c.2403_2406delCCTT	p.Leu802Alafs*126	N
E17	c.2416_2417insG	p.Val806Glyfs*10	N

E: Exon; N: not in Exome Aggregation Consortium (ExAC)

Table S2 Runs of homozygosity in the proband

chrom	begin	end	length (Mb)
1	165376227	176526147	11.15
13	37569200	86369120	48.8
14	24031694	50195679	26.16
19	7166138	17919024	10.75
19	49206631	52223121	3.02
2	36668395	45619987	8.95
7	147926888	152517622	4.59
**Total**			**113**

550-950Mb: 1 ST Degree; 250-635Mb: 2nd Degree; 100-300Mb: 3rd Degree; 30-75Mb: 4th Degree;

Table S3. All variants of LDLR, PCSK9 and APOB in proband

cDNA change	Protein change	location	function	MAF	Clinical classification
LDLR					
c.940+36G>A	p.(=)	intronic		0.0419	benign
c.1056C>T	p.(=)	exonic	synonymous	0.00017	benign
c.1060+7T>C	p.(=)	intronic		0.9999	benign
c.1413A>G|	p.(=)	exonic	synonymous	0.6106	benign
c.1705+56C>T	p.(=)	intronic		0.1471	Likely benign
c.2164C>T	p.Gln722Ter	exonic	nonsense	N	pathogenic
c.2232A>G	p.(=)	exonic	synonymous	0.7498	benign
c.2389+47G>A	p.(=)	intronic		0.0609	benign
c.2548-42A>G	p.(=)	intronic		0.5287	benign
PCSK9					
c.207+15A>G	p.(=)	intronic		0.0884	benign
c.277C>T	p.Arg93Cys	exonic	missense	0.0007	Likely benign
c.657+82G>A	p.(=)	intronic		0.3752	Likely benign
c.1026A>G	p.(=)	exonic	synonymous	0.0203	benign
c.1355-56C>T	p.(=)	intronic		0.7972	benign
c.1380A>G	p.(=)	exonic	synonymous	0.1822	benign

c.1420G>A	p.Val474Ile	exonic	missense	0.1822	benign
c.2009G>A	p.Gly670Glu	exonic	missense	0.1106	benign
APOB					
c.8216C>T	p.Pro2739Leu	exonic	missense	0.1965	benign
c.6937A>G	p.Ile2313Val	exonic	missense	0.0184	benign
c.4265A>G	p.Tyr1422Cys	exonic	missense	0.0003	Likely benign
c.1853C>T	p.Ala618Val	exonic	missense	0.3655	benign
c.1830-103A>C	p.(=)	intronic		0.8745	Likely benign

(=): no change in protein; N: no report
